# Noncanonical NF-κB Signaling Upregulation in Inflammatory Bowel Disease Patients is Associated With Loss of Response to Anti-TNF Agents

**DOI:** 10.3389/fphar.2021.655887

**Published:** 2021-06-10

**Authors:** Vu Q. Nguyen, Kristin Eden, Holly A. Morrison, Megan B. Sammons, Kristin K. Knight, Siena Sorrentino, Rebecca M. Brock, Douglas J. Grider, Irving C. Allen, Dario Sorrentino

**Affiliations:** ^1^ IBD Center, Division of Gastroenterology, Virginia Tech Carilion School of Medicine, Roanoke, VA, United States; ^2^ Department of Biomedical Sciences and Pathobiology, Virginia-Maryland College of Veterinary Medicine, Virginia Tech, Blacksburg, VA, United States; ^3^ Department of Basic Science Education, Virginia Tech Carilion School of Medicine, Roanoke, VA, United States; ^4^ Graduate Program in Translational Biology, Medicine, and Health, Virginia Tech, Blacksburg, VA, United States; ^5^ Department of Pathology, Virginia Tech Carilion School of Medicine, Roanoke, VA, United States; ^6^ Department of Clinical and Experimental Medical Sciences, University of Udine School of Medicine, Udine, Italy

**Keywords:** noncanonical NF-κB pathway, anti-TNF agents, inflammatory bowel disease, Crohn’s disease, ulcerative colitis, alternative pathway, NIK, therapeutic response

## Abstract

**Objectives:** Targeting tumor necrosis factor (TNF) with biologic agents, such as infliximab and adalimumab, is a widely used and effective therapeutic strategy in inflammatory bowel disease (IBD). Unfortunately, a significant number of patients fail to respond or lose response over time to these agents. Previous studies have defined multiple complex roles for canonical NF-κB signaling in the pathogenesis of IBD. However, preliminary evidence suggests that the lesser defined noncanonical NF-κB signaling pathway also contributes to disease pathogenesis and response to anti-TNF agents. The objective of this study was to evaluate this hypothesis in Crohn’s disease (CD) and ulcerative colitis (UC) patients.

**Design:** A total of 27 subjects with IBD (19 with CD and 8 with UC) and 15 control subjects were tested. Clinical criteria, patient history, and endoscopic disease activity were factors used to categorize patients and define therapeutic response. Biopsy specimens were collected during colonoscopy and expression was determined for 88 target genes known to be associated with noncanonical NF-κB signaling and IBD.

**Results:** Noncanonical NF-κB signaling was significantly upregulated in IBD patients and was associated with increased gastrointestinal inflammation, epithelial cell death, lymphocyte migration, and Nod-like receptor signaling. Furthermore, noncanonical NF-κB signaling was further upregulated in patients unresponsive to anti-TNF agents and was suppressed in responsive patients. *MAP3K14, NFKB2, CCL19, CXCL12*, and *CXCL13* were significantly dysregulated, as were genes that encode pathway regulators, such as *CYLD, NLRP12,* and *BIRC2/3*.

**Conclusion:** Our study identifies a previously uncharacterized role for the understudied noncanonical NF-κB signaling pathway in the pathogenesis of IBD and anti-TNF therapy responsiveness. The genes and pathways identified may ultimately prove useful in IBD management and could potentially be used as biomarkers of drug response.

## Introduction

Inflammatory bowel diseases (IBD)–Crohn’s disease (CD) and ulcerative colitis (UC)-are characterized by chronic inflammation of the gastrointestinal tract, driven by elements of both the innate and adaptive immune systems in genetically susceptible individuals. Together these diseases afflict approximately over 6 million people worldwide, which makes them a significant global health and economic burden (Ng et al., 2018). The nuclear factor kappa B (NF-κB) family of transcription factors are master regulators of inflammation and drive a diverse spectrum of biological processes (Rothschild et al., 2018). This signaling cascade is commonly found dysregulated in IBD patients, leading to dysfunctional cytokine and chemokine production in the gastrointestinal tract. NF-κB signaling occurs through two distinct pathways, defined as the canonical pathway and the noncanonical (or alternative) pathway.

In the context of IBD, the overwhelming majority of studies have focused on the canonical NF-κB signaling cascade. This cascade relies on the proteins RelA (p65) and p50 which form the heterodimer RelA/p50. Upon activation of this pathway, this heterodimer translocates into the nucleus, where it functions as a transcription factor. In this pathway, signaling is rapid and constitutive. Activation of the cascade results in the transcription of a wide range of well characterized inflammatory mediators such as IL-1β, TNF, and IL-6 (McDaniel et al., 2016). TNF in particular plays a central role in the pathogenesis of IBD (Billmeier et al., 2016) as shown by the clinical efficacy of anti-TNF medications such as infliximab (IFX) or adalimumab (ADA)-mainstay therapies for both CD and UC. These therapies have been shown to reduce the risk of hospitalization, surgery, and improve quality of life (Mao et al., 2017). In addition, they have been shown to prevent CD relapse after surgery (Sorrentino 2013). However, up to 40% of IBD patients do not respond to initial treatment with anti-TNF agents [primary nonresponse (PNR)], and a similar proportion of patients lose response over time [secondary loss of response (SLR)] after initially achieving clinical remission (Danese et al., 2011; Ben-Horin et al., 2014). The mechanism of PNR and SLR to anti-TNF agents is not completely understood and likely to be multifactorial. Several explanations have been suggested, including the immune system switch to an alternative inflammatory pathway, immunogenicity with production of drug neutralizing antibodies and medical unresposiveness due to advanced disease (Sorrentino 2008; Yarur et al., 2014; Biancheri et al., 2015; Brandse et al., 2016). While one mechanism does not exclude others and they might all play a role in different circumstances, the possibility of alternative pathways driving inflammation in IBD may provide insight into why medications targeting molecules different from TNF might be effective in SLR and PNR.

Unlike the canonical pathway, there is minimal data on the role of this signaling cascade in IBD. Noncanonical NF-κB signaling is highly regulated at the post-transcriptional and post-translational level, resulting in a slower, more controlled, and chronic signaling response. The noncanonical pathway relies on a heterodimer different from that of the canonical pathway and is comprised of RelB and 100, which is later cleaved into its active form of RelB and p52 (Sun 2011). In the noncanonical signaling cascade, NF-κB initiates the transcription of a limited repertoire of target genes, including those coding for the chemokines CXCL12, CXCL13, CCL19, and CCL21 (Supplementary Figure S1). Prior research from our group using a pre-clinical mouse model found that mice lacking negative regulators of noncanonical NF-κB signaling - such as the Nod-like receptor NLRP12-demonstrated increased noncanonical pathway activation and susceptibility to models of IBD and inflammation-driven colon tumorigenesis (Allen et al., 2012). These findings suggest a role of noncanonical NF-κB signaling in IBD pathogenesis. In this study, we examine the hypothesis that this pathway may be involved in anti-TNF agents response in IBD patients.

## Materials and Methods

### Protection of Human Subjects

All studies were conducted following the regulations, policies, and guidelines set forth by the National Institutes of Health for research involving human subjects. All studies were conducted under the approval of the Institutional Review Board of Carilion Clinic and Virginia Tech Carilion School of Medicine.

### Patient Selection

A total of 27 established IBD patients (19 with CD and 8 with UC) and 15 control subjects were prospectively recruited from the IBD Center at Carilion Clinic from 2015 to 2018. Patients were ≥ 18 years of age, without other gastrointestinal or systemic disorders, without recent NSAIDs use, and scheduled for colonoscopy for clinical indications. Pregnant patients were excluded.

### Sample Collection

Tissue samples from each patient were obtained through biopsies of both visible lesions and non-affected areas in the colon and/or the terminal ileum. Tissue samples were stored in RNAlater at −80°C. Patients were separated into different groups based on diagnosis (CD or UC), type of medication use, and response to therapy. Tissue from controls was obtained from patients undergoing colonoscopy for reasons other than IBD (e.g., rectal bleeding) and included in the study only after endoscopy and histology proved to be normal.

### Data Collection

Clinical data were abstracted from the patient medical charts. Demographics variables collected included age, gender, and smoking status. For IBD patients, disease diagnosis, and phenotype were determined at the time of study enrollment based on the Montreal disease classification (Satsangi et al., 2006). IBD medications and response to anti-TNF agents were recorded. Anti-TNF agents consisted of IFX and ADA in this patient cohort. Loss of response to anti-TNF therapy was classified as PNR or SLR. PNR was defined as complete lack of response to initiation of anti-TNF therapy whereas patients with SLR were those who initially responded to an anti-TNF agent - at least through the induction phase - but subsequently lost response. Clinical symptoms, inflammatory markers, imaging, and/or endoscopy findings were used to determine response.

### Tissue Processing and Data Analysis

Biopsy samples were removed from RNAlater, finely minced, and homogenized in RLT buffer (Qiagen) with 2-mercaptoethanol. Tissues were processed using an AllPrep Kit (Qiagen) following the manufacturer’s protocol. RNA was analyzed for quality and concentration using a NanoDrop™. Pools were made using equal concentrations of RNA from each patient sample for each experimental group. Patient samples were assigned to pools randomly and at least three pools were generated for each experimental group. The total amount of RNA used for each cDNA reaction was 600 ng and cDNA was generated using a First Strand Kit (Qiagen) following manufacturer’s protocols. Following cDNA preparation, gene expression was evaluated using a custom Qiagen RT^2^ Superarray following manufacturer’s protocol. A list of 88 candidate genes for the custom Superarray platform is provided in Supplementary Figure S2. For each array, gene expression was determined utilizing the ΔΔCt method, using a panel of five housekeeping genes and internal controls provided on each array. Gene expression was determined using a 7,500 Fast Block Real-Time PCR System (ABI). Data was analyzed using SA Biosciences Data Analysis Center and Ingenuity Pathway Analysis (IPA). IPA images were then generated using BioRender.

In addition to the analysis of pooled samples, quantitative rtPCR was also performed in select genes of interest for each individual specimen. Complimentary DNA was generated from each specimen using an ABI High Capacity cDNA kit, in accordance with the manufacturer’s protocol and 1 μg amplified using the Taqman-based rtPCR platform (ThermoFisher) on the ABI system. Gene expression was determined using the ΔΔCt method. All data were normalized to 18s and the fold change in gene expression was determined.

### Statistical Analysis

For gene expression analysis, data was analyzed using GraphPad Prism, version 6 (GraphPad Software, Inc., San Diego, CA). Student’s two-tailed t test was used for comparison between experimental groups. Multiple comparisons were conducted using one-way and two-way ANOVA where appropriate followed by Tukey post-test for multiple pairwise examinations. Correlation was computed using GraphPad Prism. Changes were identified as statistically significant if *p*-value was less than 0.05. Mean values were reported together with the SEM. The number of patients were determined based on a power analysis conducted using findings from a retrospective metadata analysis of publicly accessible gene expression data from dataset GSE16879. Gene expression was determined for a single marker of noncanonical NF-κB signaling (CXCL13) and the average and standard deviation of expression for this gene was used to determine the number of patients to target in the clinical study presented here.

## Results

### Noncanonical NF-κB is Upregulated in IBD

Tissues samples were obtained from 27 IBD patients and 15 controls. In the IBD group, 19 patients had CD and eight patients had UC (Table 1). The mean age of IBD patients was 41 years, 44% were females and 15% were active smokers. Of this patient pool, 33% of IBD patients were anti-TNF responders while 44% were not on medications or were taking medications other than anti-TNF agents. All anti-TNF non-responders in this study experienced SLR. The majority of CD and UC patients had ileocolonic and extensive colonic involvement respectively.

**TABLE 1:** Patient characteristics.

	IBD	Controls
N	27	15
Age (years)	41 ± 15	55 ± 14
Female	12 (44%)	6 (40%)
Smoker	4 (15%)	2 (13%)
CD	19 (70%)	
**CD extent**		
L1 (ileal)	5 (26%)	
L2 (colonic)	7 (37%)	
L3 (ileocolonic)	7 (37%)	
**UC extent**		
E1 (rectum)	2 (25%)	
E2 (left sided)	2 (25%)	
E3 (extensive)	4 (50%)	
**IBD medications**		
Anti-TNF agents	15 (56%)	
Non-anti-TNF therapies	10 (37%)	
No therapy	2 (7%)	
Anti-TNF responsive	5/15 (33%)	

Age is reported as mean ± SD.

**FIGURE 1:**
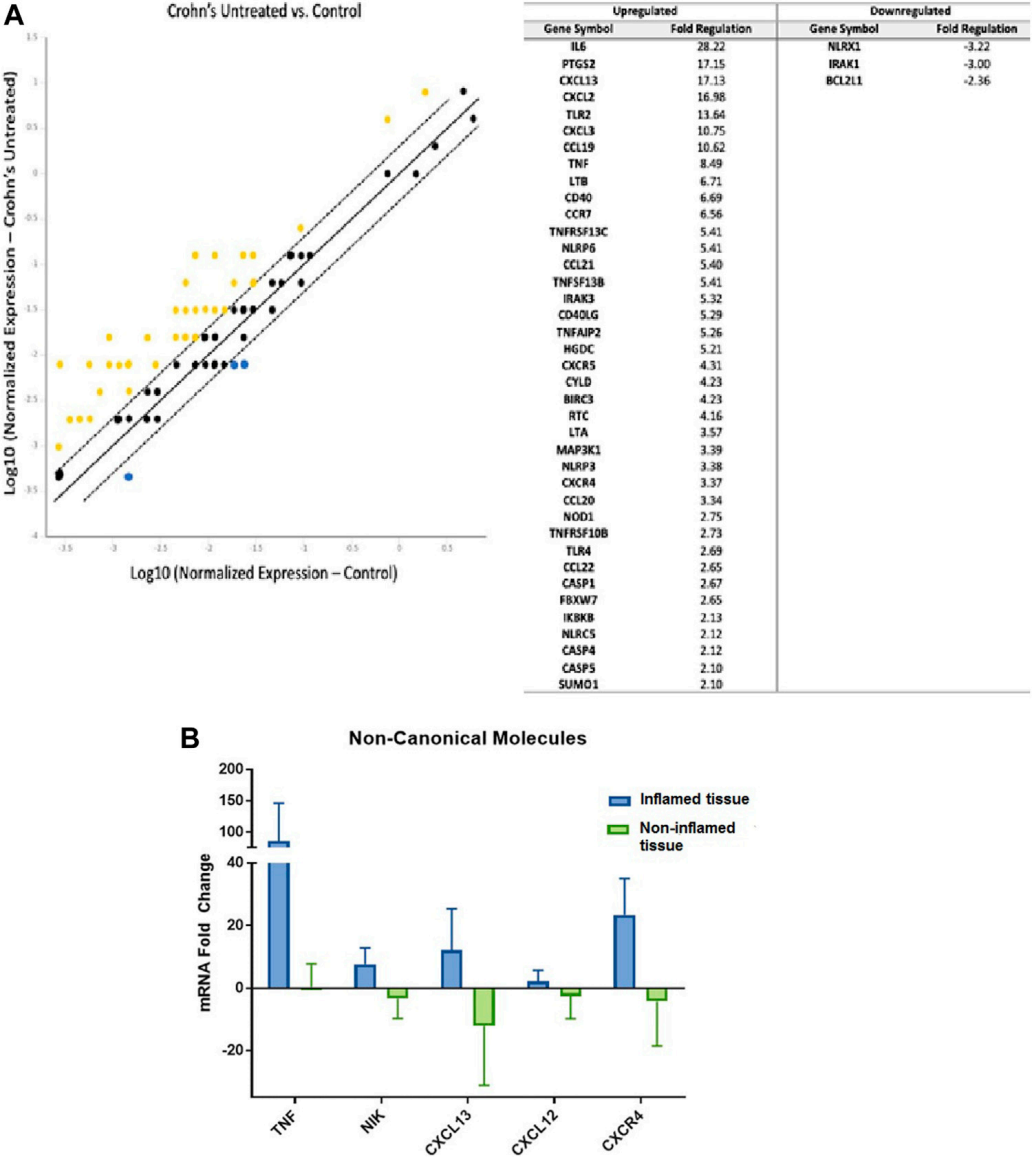
Noncanonical NF-κB Signaling is Upregulated in Inflammatory Bowel Disease Patients **(A)** Left: Compared to gene expression from intestinal tissues of controls (*n* = 15, 3 pools), 39 genes related to noncanonical NF-κB pathway were upregulated in intestinal tissues obtained from untreated CD patients (*n* = 2, 1 pool) with yellow indicating upregulation and blue indicating downregulation (lines represent 1, 0, and −1 fold change; genes outside of dashed lines are considered significantly dysregulated). Housekeeping genes used for normalization include *ACTB, B2M,* and *GADPH;* Right: list of 39 upregulated genes and three downregulated genes and fold change values. **(B)**. Using real-time PCR of individual patient samples, the main noncanonical NF-kB pathway components TNF, NIK, CXCL12, CXCL13, and CXCR4 were found to be upregulated in inflamed areas compared to non-inflamed areas obtained from the same individual patients (inflamed lesions *n* = 4; non-inflamed tissue *n* = 4).

First, we examined the association of the noncanonical NF-κB pathway in IBD patients compared to controls. We found a significant upregulation of 39 genes and downregulation of three genes associated with noncanonical NF-κB signaling in the pooled untreated CD patients (Figure 1A). These dysregulated genes encode for proteins with crucial roles in the signaling cascade. We also conducted individual real-time PCR analysis on a selection of these genes to validate the findings of the gene expression arrays. Individual qPCR was conducted on five genes that were significantly dysregulated in the custom gene expression arrays and are critical points in the noncanonical NF-κB signaling pathway (Supplementary Figure S1). TNF is one of the activating molecules; NIK is a critical kinase required for perpetuation of this activating signal; and CXCL12, CXCL13, and CXCR4 are products of the signaling pathway. The individual gene expression was consistent with custom arrays: *TNF*, *MAP3K14* (NIK), *CXCL12*, *CXCL13*, and *CXCR4* genes were all significantly upregulated in biopsies collected from inflamed areas compared to biopsies collected from non-inflamed areas in the same individual patients (Figure 1B).

### Noncanonical NF-κB Signaling is Downregulated in Anti-TNF Responsive Patients

**FIGURE 2:**
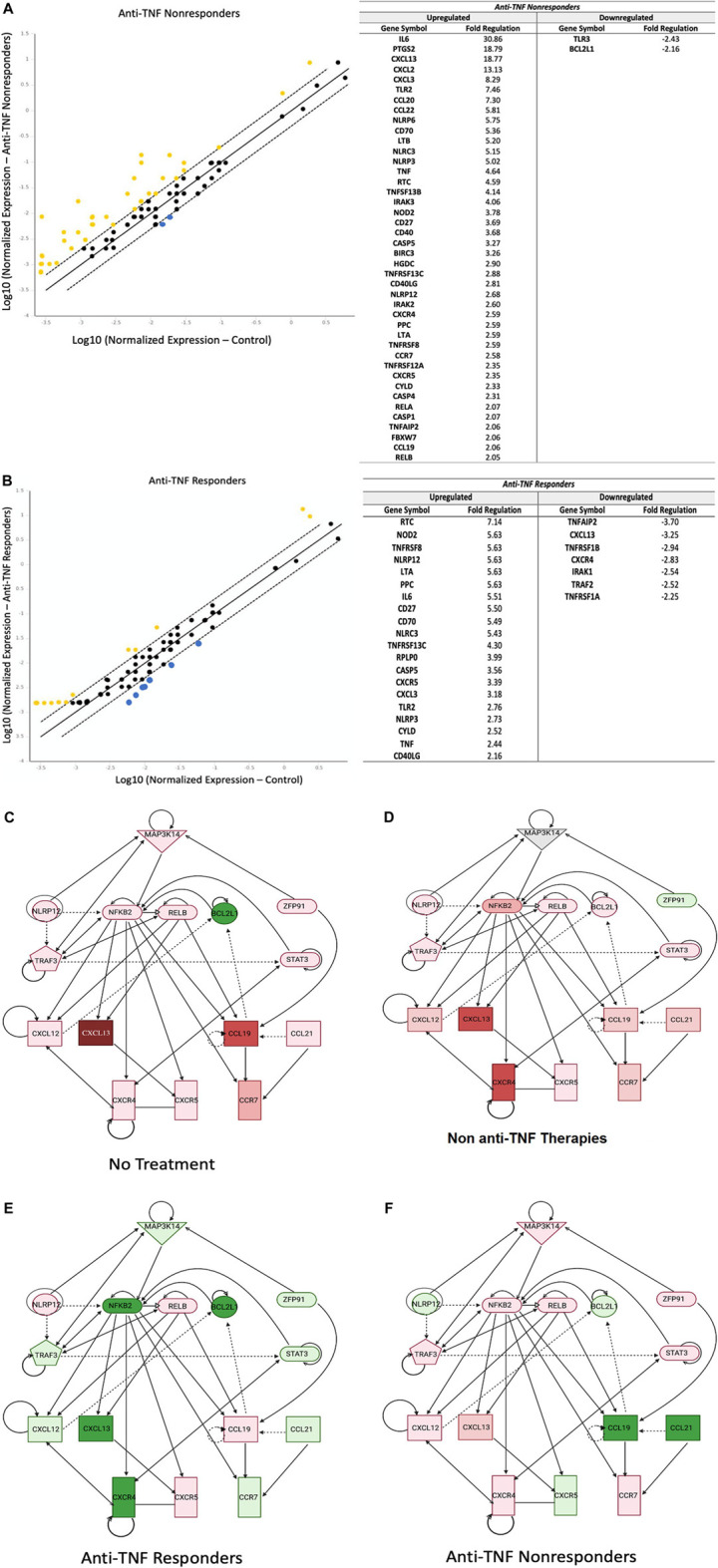
Noncanonical NF-κB is Downregulated in IBD Patients Responding To Anti-TNF Therapy. **(A)**: Inflamed tissues from anti-TNF nonresponders (*n* = 10, two pools, CD patients and UC patients) showed 39 upregulated genes related to the noncanonical NF-κB signaling, compared to only 19 genes in anti-TNF responders (*n* = 5, two pools) **(B)**, with yellow indicating upregulation and blue indicating downregulation (lines represent 1, 0, and −1 fold change; genes outside of dashed lines are considered significantly dysregulated). A list of the involved genes is provided next to each graph. Housekeeping genes used for normalization include *ACTB, B2M,* and *GADPH* **(C–F)**: Ingenuity Pathway Analysis revealed that noncanonical NF-κB was a significant signaling hub that was downregulated in patients responding to anti-TNF therapy. Expression levels of the main noncanonical genes *NIK (MAP3K14), CXCL12, CXCL13*, and *CCL21* were upregulated in both untreated patients (*n* = 2, one pool) **(C)** and patients treated with non-anti-TNF medications (*n* = 10, two pools) **(D)**. However, anti-TNF responders (*n* = 5, two pools) **(E)**, showed a distinct attenuation of expression of these genes. Meanwhile, anti-TNF non-responders showed upregulation of *CXCL12* and *CXCL13* and downregulation of *CCL21*. Note: green represents downregulation; red represents upregulation.

We then examined the role of the noncanonical NF-κB pathway in IBD patients in relation to their responsiveness to anti-TNF agents. Compared to controls, in IBD patients not responding to anti-TNF therapy, we identified 42 upregulated genes and only two downregulated genes (Figure 2A). Notably, CXCL13 was significantly upregulated (+18.77 fold) in nonresponders.

Conversely, in IBD patients responding to anti-TNF agents we identified 20 upregulated genes and seven downregulated genes involved in noncanonical NF-κB signaling (Figure 2B). Upregulated genes included *NLRP12*, a negative regulator of noncanonical signaling, while downregulated genes included noncanonical chemokine *CXCL13* and noncanonical receptor for *CXCR4*.

Pathway analysis revealed significant downregulation of noncanonical NF-κB signaling in IBD patients responsive to anti-TNFα therapy compared to untreated IBD patients and IBD patients treated with other (non-anti-TNF) medications (Figures 2C–F). This decreased expression was observed in several genes throughout the signaling pathway, and specifically included reduced expression of *MAP3K14, NFKB2, TRAF3, STAT3, CXCL12, CXCL13, CXCR4*, and *CCR7* that were not observed in the other IBD patient groups. Interestingly, in IBD patients unresponsive to anti-TNF therapy, noncanonical NF-κB signaling was upregulated compared to untreated IBD patients. In these patients, upregulation of noncanonical NF-κB signaling was strongly associated with the downregulation of *NLRP12* and upregulation of *MAP3K14, CXCL12,* and *CXCL13.* Together, these data demonstrate that noncanonical NF-κB signaling was significantly decreased in anti-TNF responders and increased in anti-TNF nonresponders.

### CXCL12 and CXCL13 Expression is Correlated to Anti-TNF Responsiveness

**FIGURE 3:**
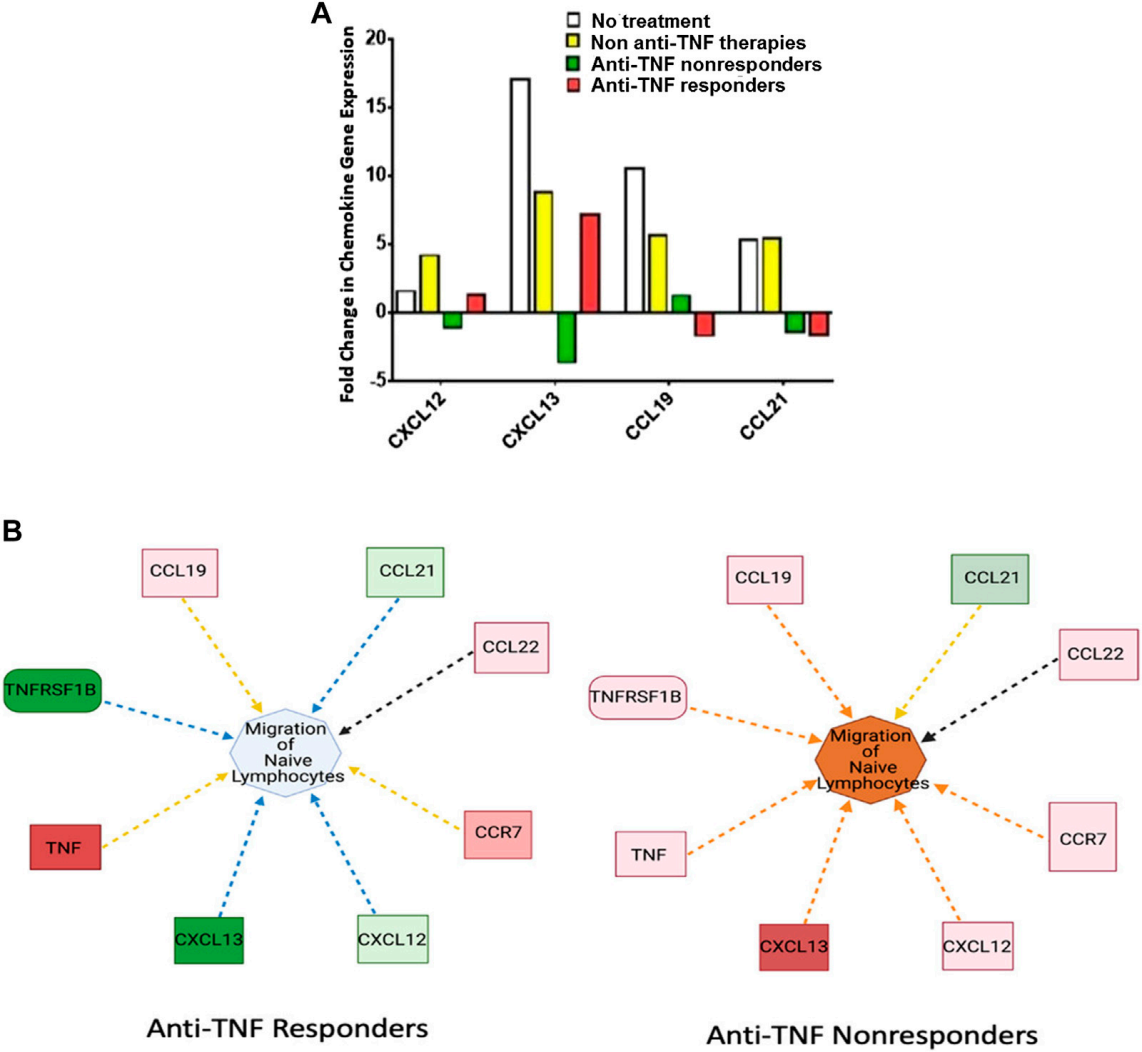
CXCL12 and CXCL13 are Downregulated in IBD Patients Responding to Anti-TNFα Therapy. **(A)** CXCL12, CXCL13, CCL19, and CCL21 were significantly upregulated in both untreated IBD patients (*n* = 2, one pool) and IBD patients treated with non-anti-TNF medications (*n* = 10, two pools). CXCL12, CXCL13, and CCL19 displayed opposite expression profiles based on anti-TNF responsiveness, with upregulation in anti-TNF nonresponders (*n* = 10, two pools) and downregulation in responders (*n* = 5, two pools) for CXCL12 and CXCL13. CCL21 remained downregulated regardless of response to anti-TNF agents. **(B)** Predicted effect on the migration of naïve lymphocytes to inflamed tissue based on changes of the noncanonical chemokines which are involved in lymphocyte trafficking. In anti-TNF responders, lymphocyte trafficking is reduced. By contrast anti-TNF nonresponders demonstrate increased lymphocyte trafficking. In general, the increase in noncanonical signaling molecules results in a large increase in predicted lymphocyte migration. Note: Blue and orange dashed lines with arrows indicate indirect inhibition and activation, respectively. Yellow and black dashed lines with arrows depict inconsistent effects and no prediction, respectively.

Currently, only four major chemokines have been associated with the noncanonical NF-κB signaling cascade, CCL19, CCL21, CXCL12, and CXCL13. We examined these chemokine expression patterns in relation to anti-TNF responsiveness. In untreated IBD patients and in those treated with other (non-anti-TNF) therapies, *CCL19, CCL21, CXCL12, and CXCL13* expression were upregulated. IBD patients responding to anti-TNF therapy demonstrated a significant decrease in *CXCL12, CXCL13*, and *CCL21* compared to those in the untreated IBD group (Figure 3A). By contrast, *CXCL12* and *CXCL13* were significantly upregulated in IBD patients not responding to anti-TNF therapy while *CCL19* and *CCL21* were downregulated in these patients. Both *CXCL12* and *CXCL13* were strongly correlated with anti-TNF responsiveness, whereas *CCL19* and *CCL21* only demonstrated a weak correlation. Based on the expression profile associated with these chemokines, it appears that migration of naïve lymphocytes was the signaling network most strongly altered in patients not responding to anti-TNF agents (Figure 3B). Hence, by contrast with nonresponders, in IBD patients responding to anti-TNF therapy, the downregulation of *CXCL12* and *CXCL13* could result in reduced migration of lymphocytes to inflamed tissues.

## Discussion

Anti-TNF therapy acting through the canonical NF-κB signaling pathway has been the cornerstone of IBD treatment for the past several years. Despite its success, a significant proportion of IBD patients may not respond or lose response over time to anti-TNF therapy. This population of anti-TNF nonresponders has an increased risk of disease complications but might respond to newer biologic medications such as ustekinumab or vedolizumab (Feagan et al., 2016 and Feagan et al.2017). The mechanisms of PNR and SLR to anti-TNF agents are poorly understood. One possible explanation is that the driving mechanism of inflammation in these IBD patients may have shifted to an alternative signaling pathway.

The noncanonical NF-κB pathway has been recognized as a key regulator and promoter of the adaptive immune system, particularly in the development of secondary lymphoid organs. Prior studies from our group have demonstrated the role of this signaling pathway in mouse models of colon inflammation and eosinophilic esophagitis (Allen et al., 2012; Eden et al., 2017).

In this study, we found significant upregulation of the genes involved in the noncanonical NF-κB pathway in IBD patients compared to healthy controls. Furthermore, the main chemokines in this pathway, namely CXCL12 and CXCL13, were significantly downregulated in IBD patients who responded to anti-TNF agents and were upregulated in nonresponders. These findings suggest that the activation of the noncanonical NF-κB signaling pathway may contribute to the mechanism underlying the lack/loss of response to anti-TNF therapy. The upregulated genes in the noncanonical NF-κB pathway involved in lack/loss of response to anti-TNF therapy may involve leukocyte migration to the affected tissue as predicted by IPA analysis.

The relatively small sample size and single center nature are limitations of our study. In addition, it is unclear how many of our anti-TNF nonresponders would respond to additional anti-TNF agents (Swaminath et al., 2009; Löfberg et al., 2012). Whether the expression pattern of the noncanonical genes differs in patients who respond to a second anti-TNF agent compared to those who do not respond to any anti-TNF medication remains unknown. Also, our study does not clarify the role of this pathway in PNR–since all of our patients experienced SLR.

In conclusion, this is the first study in humans that has explored the role of the noncanonical NF-κB signaling in the pathogenesis of IBD. More importantly, our findings provide a potential mechanistic insight into the lack/loss of response to anti-TNF agents in IBD. If our observations are confirmed, targeting the noncanonical NF-κB signaling pathway could significantly impact IBD management and therapy outcomes.

## Data Availability

The authors acknowledge that the data presented in this study must be deposited and made publicly available in an acceptable repository, prior to publication. Frontiers cannot accept a article that does not adhere to our open data policies.

## Supplementary Material

SUPPLEMENTARY FIGURE 1 Schematic Illustration of Key Components of Noncanonical NF-κB Signaling Significantly Dysregulated in IBD Patients. Our data revealed a significant increase in chemokines associated with noncanonical NF-κB signaling, including CXCL12, CXCL13, CCL9, and CCL21. Additional pathway analysis revealed that these changes were associated with increased expression of genes encoding a variety of proteins that regulate the non-canonical NF-κB signaling pathway through modulation of NIK, including the up-regulation of cIAP1, cIAP2, CYLD, and NLRP12 and the down-regulation of BCL10, ZPF91, and A20. Created with BioRender.com.

SUPPLEMENTARY TABLE 1 Custom Gene Array to Evaluate Noncanonical NF-κB Signaling in IBD Patients. The gene list for the custom superarray platform is provided with RefSeq Number and Gene Symbol. These 88 gene candidates serve a role in the noncanonical NF-κB pathway and dysregulated transcription levels may have implications on IBD pathogenesis and drug responsiveness. For each array, gene expression was determined utilizing the delta-deltaCt method, using a panel of five housekeeping genes and internal controls provided on each array.
