# Supplementary material for: Noncanonical NF-κB Signaling Upregulation in Inflammatory Bowel Disease Patients is Associated With Loss of Response to Anti-TNF Agents
Source: Front Pharmacol. 2021 Jun 10;12:655887. doi: 10.3389/fphar.2021.655887 (PMC8223059; doi:10.3389/fphar.2021.655887)
Supplement: Supplementary file 1 [file Table1.pdf]

WELL	GeneBank	Symbol	Description	Gene Name
A1	NM_005163	AKT1	V-akt murine thymoma viral oncogene homolog 1	AKT, CWS6, PKB, PKB-ALPHA, PRKBA, RAC, RAC-ALPHA
A2	NM_003921	BCL10	B-cell CLL/lymphoma 10	CLAP; mE10; CIPER; IMD37; c-E10; CARMEN
A3	NM_000633	BCL2	B-cell CLL/lymphoma 2	Bcl-2; PPP1R50
A4	NM_001191	BCL2L1	BCL2 like 1	BCLX; BCL2L; Bcl-X; PPP1R52; BCL-XL/S
A5	NM_001166	BIRC2	Baculoviral IAP repeat containing 2	API1, HIAP2, Hiap-2, MIHB, RNF48, c-IAP1, cIAP1
A6	NM_001165	BIRC3	Baculoviral IAP repeat containing 3	AIP1, API2, CIAP2, HAIP1, HIAP1, MALT2, MIHC, RNF49, c-IAP2
A7	NM_033292	CASP1	Caspase 1, apoptosis-related cysteine peptidase (interleukin 1, beta, convertase)	ICE, IL1BC, P45
A8	NM_001225	CASP4	Caspase 4 (human homolog of mouse casp11)	ICE(rel)II, ICEREL-II, ICH-2, Mih1/TX, TX
A9	NM_001136109	CASP5	Caspase 5, human homology of mouse casp11)	ICH-3; ICEREL-III; ICE(rel)III
A10	NM_006274	CCL19	C-C motif chemokine 19	ELC; CKb11; MIP3B; MIP-3b; SCYA19
A11	NM_004591	CCL20	Chemokine (C-C motif) ligand 20	CKb4, Exodus, LARC, MIP-3-alpha, MIP-3a, MIP3A, SCYA20, ST38
A12	NM_002989	CCL21	chemokine (C-C motif) ligand 21	ECL; SLC; CKb9; TCA4; 6Ckine; SCYA21
B1	NM_002990	CCL22	Chemokine (C-C motif) ligand 22	A-152E5.1, ABCD-1, DC, B-CK, MDC, SCYA22, STCP-1
B2	NM_002985	CCL5	Chemokine (C-C motif) ligand 5	D17S136E, RANTES, SCYA5, SIS-delta, SISd, TCP228, eoCP
B3	NM_001301714	CCR7	chemokine (C-C motif) receptor 7	BLR2; EBI1; CCR-7; CD197; CDw197; CMKBR7; CC-CKR-7
B4	NM_001242	CD27	CD27 molecule	T14; S152; Tp55; TNFRSF7; S152. LPFS2
B5	NM_001250	CD40	CD40 molecule, TNF receptor superfamily member 5	Bp50, CDW40, TNFRSF5, p50
B6	NM_000074	CD40LG	CD40 ligand	IGM; IMD3; TRAP; gp39; CD154; CD40L; HIGM1; T-BAM; TNFSF5; hCD40L
B7	NM_001252	CD70	CD70 molecule, tumor necrosis factor (ligand) superfamily, member 7	CD27L; CD27LG; TNFSF7; TNLG8A
B8	NM_001278	CHUK	Conserved helix-loop-helix ubiquitous kinase	IKBKA, IKK-alpha, IKK1, IKKA, NFKBIKA, TCF16
B9	NM_000609	CXCL12	chemokine (C-X-C motif) ligand 12	IRH; PBSF; SDF1; TLSF; TPAR1; SCYB12
B10	NM_001142523	IRAK3	**interleukin 1 receptor associated kinase 3**	**ASRT5; IRAKM**
B11	NM_006419	CXCL13	chemokine (C-X-C motif) ligand 13	BLC; BCA1; ANGIE; BCA-1; BLR1L; ANGIE2; SCYB13
B12	NM_001114182	IRAK4	**interleukin 1 receptor associated kinase 4**	**IPD1; REN64; IRAK-4; NY-REN-64**
C1	NM_002089	CXCL2	Chemokine (C-X-C motif) ligand 2	CINC-2a, GRO2, GROb, MGSA-b, MIP-2a, MIP2, MIP2A, SCYB2
C2	NM_002090	CXCL3	Chemokine (C-X-C motif) ligand 3	CINC-2b, GRO3, GROg, MIP-2b, MIP2B, SCYB3
C3	NM_001008540	CXCR4	Chemokine (C-X-C motif) receptor 4	FB22; HM89; LAP3; LCR1; NPYR; WHIM; CD184; LAP-3; LESTR; NPY3R; NPYRL; WHIMS;
C4	NM_001716	CXCR5	Chemokine (C-X-C motif) receptor 5	BLR1; CD185; MDR15. Receptor for cxcl12/13
C5	NM_001042355	CYLD	Cylindromatosis (turban tumor syndrome)	EAC; MFT; SBS; TEM; BRSS; CDMT; MFT1; CYLD1; CYLDI; USPL2
C6	NM_005228	EGFR	Epidermal growth factor receptor	ERBB, ERBB1, HER1, NISBD2, PIG61, mENA
C7	NM_001013415	FBXW7	F-box and WD repeat domain containing 7	AGO; CDC4; FBW6; FBW7; hAgo; FBX30; FBXW6; SEL10; hCdc4; FBXO30; SEL-10
C8	NM_006597	HSPA8	Heat shock cognate protein 70	Hsc70 or Hsp73, HSPA8
C9	NM_001556	IKBKB	Inhibitor of kappa light polypeptide gene enhancer in B-cells, kinase beta	IKK-beta, IKK2, IKKB, IMD15, NFKBIKB
C10	NM_003639	IKBKG	Inhibitor of kappa light polypeptide gene enhancer in B-cells, kinase gamma	AMCBX1, FIP-3, FIP3, Fip3p, IKK-gamma, IMD33, IP, IP1, IP2, IPD2, NEMO, ZC2HC9
C11	NM_000600	IL6	Interleukin 6 (interferon, beta 2)	BSF2, HGF, HSF, IFNB2, IL-6
C12	NM_001569	IRAK1	Interleukin-1 receptor-associated kinase 1	IRAK, pelle
D1	NM_001570	IRAK2	Interleukin-1 receptor-associated kinase 2	IRAK-2
D2	NM_000595	LTA	Lymphotoxin alpha (TNF superfamily, member 1)	LT, TNFB, TNFSF1
D3	NM_002341	LTB	Lymphotoxin beta (TNF superfamily, member 3)	TNFC, TNFSF3, p33
D4	NM_002342	LTBR	Lymphotoxin beta receptor (TNFR superfamily, member 3)	CD18, D12S370, LT-BETA-R, TNF-R-III, TNFCR, TNFR-RP, TNFR2-RP, TNFR3, TNFRSF3
D5	NM_173844	MALT1	Mucosa associated lymphoid tissue lymphoma translocation gene 1	IMD12, MLT, MLT1
D6	NM_005921	MAP3K1	Mitogen-activated protein kinase kinase kinase 1	MAPKKK1, MEKK, MEKK 1, MEKK1, SRXY6
D7	NM_003954	MAP3K14	mitogen-activated protein kinase kinase kinase 14	NIK
D8	NM_002468	MYD88	Myeloid differentiation primary response gene (88)	MYD88D
D9	NM_003998	NFKB1	Nuclear factor of kappa light polypeptide gene enhancer in B-cells 1	EBP-1, KBF1, NF-kB1, NF-kappa-B, NF-kappaB, NFKB-p105, NFKB-p50, NFkappaB, p105, p50
D10	NM_002502	NFKB2	Nuclear factor of kappa light polypeptide gene enhancer in B-cells 2 (p49/p100)	CVID10, H2TF1, LYT-10, LYT10, NF-kB2, p100, p52
D11	NM_020529	NFKBIA	Nuclear factor of kappa light polypeptide gene enhancer in B-cells inhibitor, alpha	IKBA, MAD-3, NFKBI
D12	NM_004556	NFKBIE	Nuclear factor of kappa light polypeptide gene enhancer in B-cells inhibitor, epsilon	IKBE
E1	NM_178844	NLRC3	NLR family, CARD domain containing 3	NOD3; CLR16.2
E2	NM_032206	NLRC5	NLR family, CARD domain containing 5	NOD4; NOD27; CLR16.1
E3	NM_001033053	NLRP1	NLR family, pyrin domain containing 1	NAC; CARD7; CIDED; NALP1; SLEV1; DEFCAP; PP1044; VAMAS1; CLR17.1; DEFCAP-L/S
E4	NM_001277126	NLRP12	NLR family, pyrin domain containing 12	RNO; PAN6; RNO2; FCAS2; NALP12; PYPAF7; CLR19.3
E5	NM_001079821	NLRP3	NLR family, pyrin domain containing 3	AII; AVP; FCU; MWS; FCAS; CIAS1; FCAS1; NALP3; C1orf7; CLR1.1; PYPAF1; AGTAVPRL
E6	NM_001276700	NLRP6	NLR family, pyrin domain containing 6	AVR; NAVR; PAN3; NALP6; PYPAF5; CLR11.4; NAVR/AVR
E7	NM_001282143	NLRX1	NLR family member X1	NOD5; NOD9; NOD26; DLNB26; CLR11.3
E8	NM_006092	NOD1	Nucleotide-binding oligomerization domain containing 1	CARD4, CLR7.1, NLRC1
E9	NM_001293557	NOD2	nucleotide binding oligomerization domain containing 2	CD; ACUG; BLAU; IBD1; NLRC2; NOD2B; CARD15; CLR16.3; PSORAS1
E10	NM_000963	PTGS2	Prostaglandin-endoperoxide synthase 2 (prostaglandin G/H synthase and	COX-2, COX2, GRIPGHS, PGG, HS, PGHS-2, PHS-2, hCox-2
E11	NM_002908	REL	V-rel reticuloendotheliosis viral oncogene homolog (avian)	C-Rel
E12	NM_021975	RELA	V-rel reticuloendotheliosis viral oncogene homolog A (avian)	NFKB3, p65
F1	NM_006509	RELB	V-rel reticuloendotheliosis viral oncogene homolog B	I-REL, IREL, REL-B
F2	NM_003804	RIPK1	Receptor (TNFRSF)-interacting serine-threonine kinase 1	RIP, RIP1
F3	NM_003150	STAT3	Signal transducer and activator of transcription 3 (acute-phase response	ADMIO, APRF, HIES
F4	NM_001293197	STUB1	STIP1 homology and U-box containing protein 1, E3 ubiquitin protein ligase	CHIP
F5	NM_001005781	SUMO1	small ubiquitin-like modifier 1	DAP1; GMP1; PIC1; SMT3; UBL1; OFC10; SENP2; SMT3C; SMT3H3
F6	NM_003264	TLR2	Toll-like receptor 2	CD282, TIL4
F7	NM_003265	TLR3	Toll-like receptor 3	CD283, IIAE2
F8	NM_138554	TLR4	Toll-like receptor 4	ARMD10, CD284, TLR-4, TOLL
F9	NM_000594	TNF	Tumor necrosis factor	DIF, TNF-alpha, TNFA, TNFSF2
F10	NM_006291	TNFAIP2	Tumor necrosis factor, alpha-induced protein 2	B94, EXOC3L3
F11	NM_006290	TNFAIP3	Tumor necrosis factor, alpha-induced protein 3	A20, OTUD7C, TNFA1P2
F12	NM_003844	TNFRSF10A	Tumor necrosis factor receptor superfamily, member 10a	APO2, CD261, DR4, TRAILR-1, TRAILR1
G1	NM_003842	TNFRSF10B	Tumor necrosis factor receptor superfamily, member 10b	CD262, DR5, KILLER, KILLER, DR5, TRAIL-R2, TRAILR2, TRICK2, TRICK2A, TRICK2B, TRICKB,
G2	NM_003839	TNFRSF11A	Tumor necrosis factor receptor superfamily member 11a	RANK
G3	NM_016639	TNFRSF12A	Tumor necrosis factor receptor superfamily member 12A	FN14
G4	NM_052945	TNFRSF13C	Tumor necrosis factor receptor superfamily member 13C	BAFFR
G5	NM_001065	TNFRSF1A	Tumor necrosis factor receptor superfamily, member 1A	CD120a, FPF, MS5, TBP1, TNF-R, TNF-R-I, TNF-R55, TNFAR, TNFR1, TNFR1-d2, TNFR55,
G6	NM_001066	TNFRSF1B	Tumor necrosis factor receptor superfamily member 1B	
G7	NM_001243	TNFRSF8	Tumor necrosis factor receptor superfamily member 8	CD30; Ki-1; D1S166E
G8	NM_003810	TNFSF10	Tumor necrosis factor (ligand) superfamily, member 10	APO2L, Apo-2L, CD253, TL2, TRAIL
G9	NM_001145645	TNFSF13B	Tumor necrosis factor superfamily member 13b	BAFF
G10	NM_003789	TRADD	TNFRSF1A-associated via death domain	
G11	NM_021138	TRAF2	TNF receptor-associated factor 2	MGC:45012, TRAP, TRAP3
G12	NM_003300	TRAF3	TNF receptor-associated factor 3	CAP-1, CAP1, CD40bp, CRAF1, IIAE5, LAP1
H1	NM_004620	TRAF6	TNF receptor-associated factor 6	MGC:3310, RNF85
H2	NM_003809	TNFSF12	tumor necrosis factor superfamily member 12	TWEAK
H3	NM_001167	XIAP	X-linked inhibitor of apoptosis	API3, BIRC4, IAP-3, ILP1, MIHA, XLP2, hIAP-3, hIAP3
H4	NM_001197051	ZFP91	zinc finger protein 91	Associates with NIK, causes stabilization and activation of NIK. PZF; DMS-8; DSM-8; FKSG11;
H5	NM_001101	ACTB	Actin, beta	BRWS1, PS1TP5BP1
H6	NM_004048	B2M	Beta-2-microglobulin	-
H7	NM_002046	GAPDH	Glyceraldehyde-3-phosphate dehydrogenase	G3PD, GAPD, HEL-S-162eP
H8	NM_000194	HPRT1	Hypoxanthine phosphoribosyltransferase 1	HGPRT, HPRT
H9	NM_001002	RPLP0	Ribosomal protein, large, P0	L10E, LP0, P0, PRLP0, RPP0
H10	SA_00105	HGDC	Human Genomic DNA Contamination	HIGX1A
H11	SA_00104	RTC	Reverse Transcription Control	RTC
H12	SA_00103	PPC	Positive PCR Control	PPC
