# Supplementary figures and images for: Noncanonical NF-κB Signaling Upregulation in Inflammatory Bowel Disease Patients is Associated With Loss of Response to Anti-TNF Agents
Source: Front Pharmacol. 2021 Jun 10;12:655887. doi: 10.3389/fphar.2021.655887 (PMC8223059; doi:10.3389/fphar.2021.655887)

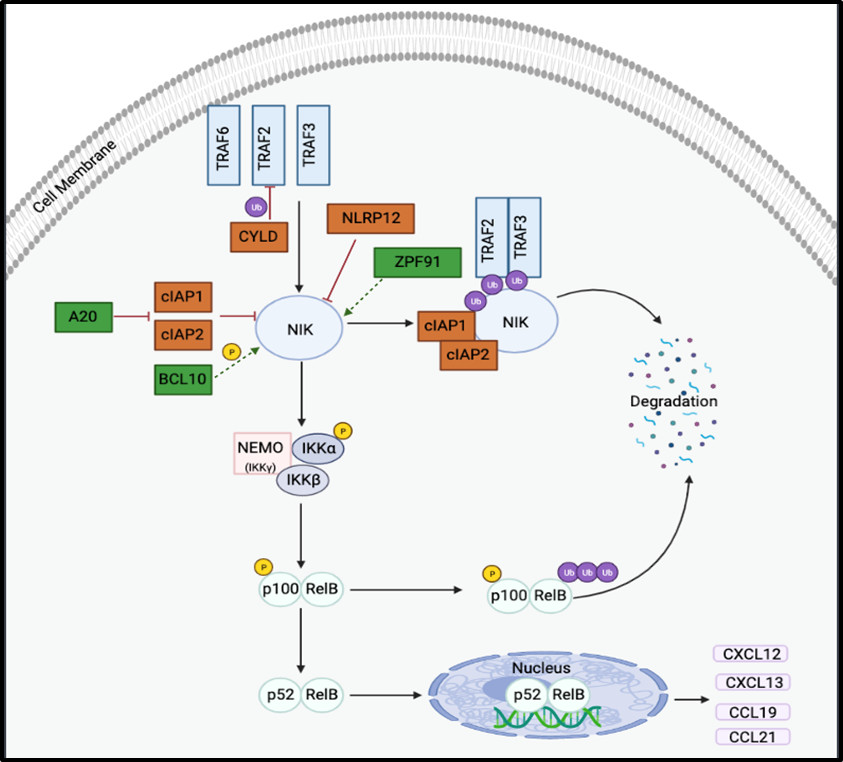

Supplement: Supplementary file 2 [file Image1.JPEG]
